# HIV vaccine development and broadly neutralizing antibodies

**DOI:** 10.1093/emph/eov004

**Published:** 2015-02-20

**Authors:** Neil S. Greenspan, Ashutosh K. Sheth, Vilok Desai

**Affiliations:** ^1^Department of Pathology, Case Western Reserve University, Cleveland, OH 44106-7288, USA and ^2^Department of Physiology and Biophysics, Case Western Reserve University, Cleveland, USA

## HUMAN IMMUNODEFICIENCY VIRUS VACCINES

Human immunodeficiency virus-1 (HIV-1) infects about 35 million people worldwide [1]. There is no vaccine in current clinical use. Although available drug treatments are generally effective in controlling infection, they are not curative, can cause significant side effects and are unable to prevent comorbidities such as cardiovascular disease and cancers. In 2012, about 1.6 million people worldwide died from acquired immunodeficiency syndrome due to HIV infection [1]. Therefore, there is global interest in developing an effective vaccine.

**Figure 1.**
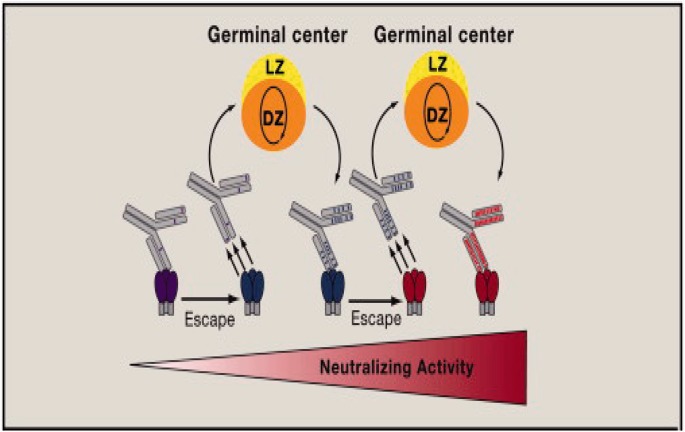
Co-evolutionary arms race between antibodies and HIV spikes. Amino acid sequence changes in HIV envelope trimers and antibody V domains are indicated by changes in color. Adapted from Ref. 7, with permission obtained through RightsLink

HIV-1 evolves extremely rapidly due to the low fidelity of the virus reverse transcriptase. So, although most HIV-1 infections start with a single virion, within a year, the HIV-1 viruses in one infected individual can exhibit about the same amount of genetic variation as is displayed by influenza A viruses globally over the course of a year [2]. Neutralizing antibodies can drive variation of HIV envelope proteins, which in turn selects for variation in antibody structure, i.e. co-evolution [3] (Figure 1). Therefore, strong protection might only be associated with vaccine immunogens capable of eliciting potent, broadly neutralizing antibodies (pbnAbs), which inactivate a very substantial fraction of HIV viruses. PbnAbs often carry an unusually high number of somatic mutations in the associated antigen-binding domains (variable or V domains) [4] and can also possess other unusual features, such as unusually long heavy chain third hypervariable regions, the most variable portion of the V domains.

## EVOLUTIONARY PERSPECTIVES

The production of pbnAbs is utterly dependent on an evolutionary process involving B lymphocytes (the only cells that physiologically make antibodies) and CD4^+^ T lymphocytes that provide critical signals to the B cells leading to proliferation and differentiation into antibody-producing plasma cells [5]. These interactions typically occur in secondary lymphoid tissues such as lymph nodes in sites known as germinal centers (GC).

A subset of the molecular signals from the T cells (e.g., the binding of CD40 ligand on the T cell to CD40 on the B cell) activate the process known as somatic hypermutation, which targets amino acid substitutions only to immunoglobulin (Ig) heavy and light chain V domains [6, 7]. The V domains are the modules that directly bind to antigens. Somatic hypermutation coupled with affinity-based selection of B cell surface Ig receptors frequently results in both increased average antibody affinity for cognate antigen (‘affinity maturation’) and enhanced breadth of neutralizing activity.

## FUTURE IMPLICATIONS

These considerations have prompted novel conceptions of vaccination, including one that employs sequential administration of a series of structurally distinguishable but similar HIV envelope proteins, as opposed to the same antigen repeatedly, as is typical for vaccination. Using a series of different immunogens is intended to guide, in a step-wise manner, the evolutionary pathways of the GC B cells towards production of pbnAbs. Investigators are actively seeking to address the many unknowns pertaining to this approach to HIV vaccine development.
